# Rapid response to chemo-immunotherapy in poorly differentiated primary tracheal squamous carcinoma with CK20 aberrance and airway obstruction: a case report

**DOI:** 10.1097/RC9.0000000000000247

**Published:** 2026-02-16

**Authors:** Rajshekhar C Jaka, Aishwarya Madasamy Swaminathan, Sunil Kumar Shetty

**Affiliations:** a Department of Surgical Oncology, Manipal Hospitals, Bengaluru, Karnataka, India; b Department of General Surgery, Kasturba Medical College, Mangalore, Karnataka, India

**Keywords:** airway obstruction, chemo-immunotherapy, CK20 aberrant expression, FDG PET/CT, PD-L1, primary tracheal carcinoma, squamous cell carcinoma

## Abstract

**Introduction and importance:**

Primary tracheal squamous cell carcinoma (SCC) is exceedingly rare and may present with acute airway obstruction, posing diagnostic and therapeutic challenges. Aberrant CK20 expression in SCC is uncommon and may mimic metastatic disease, complicating diagnosis.

**Case presentation:**

A 43-year-old male presented with sudden respiratory distress, stridor, and right supraclavicular swelling. MRI revealed a 7.9 × 6 × 6 cm tracheal wall mass with nodal involvement. Biopsy confirmed poorly differentiated SCC positive for AE1/AE3, CK5/6, p63, p40, and aberrant CK20, with PD-L1 CPS <1. lactate dehydrogenase was elevated. Urgent systemic chemotherapy (nanoparticle paclitaxel, carboplatin, 5-FU) was initiated, followed by addition of toripalimab. Marked regression was observed on fluorodeoxyglucose positron emission tomography/computed tomography, and tracheal stenting was avoided as the airway was stabilized with noninvasive ventilation. Concurrent chemoradiotherapy was subsequently administered. Despite initial response, follow up imaging revealed hepatic, pleural, and skeletal metastases.

**Clinical discussion:**

Primary tracheal SCC remains a rare and aggressive malignancy. Aberrant CK20 expression can create diagnostic pitfalls, emphasizing the importance of broad immunohistochemical profiling. Rapid response to chemo-immunotherapy, even in PD-L1 low tumors, suggests its potential role in emergent airway compromise. Nonetheless, disease progression highlights the need for vigilant surveillance and possible molecularly guided therapy.

**Conclusion:**

Early recognition and prompt initiation of chemo-immunotherapy can stabilize airway compromise and induce rapid response in tracheal SCC. However, aggressive biology mandates close follow-up and individualized, multidisciplinary management.

## Introduction

Primary tracheal malignancies are extremely rare, representing less than 0.1% of all cancers, with squamous cell carcinoma (SCC) being the most common histologic subtype in adults^[1,2]^. These tumors often present late due to nonspecific respiratory symptoms, such as cough or stridor, leading to airway compromise or nodal metastases^[1,2]^. Aberrant CK20 expression in SCC is rare but diagnostically challenging, as it can mimic gastrointestinal or urothelial primaries^[3,4]^. Accurate immunohistochemical characterization is essential for distinguishing primary tracheal tumors from metastatic lesions^[1,2,5]^.

**HIGHLIGHTS**

- A 43-year-old male presented with acute airway obstruction caused by a large primary tracheal squamous cell carcinoma – an exceptionally rare malignancy.
- Immunohistochemistry revealed aberrant CK20 expression in the tumor, a rare finding in SCC that complicated diagnostic interpretation.
- The patient was managed non-invasively with high-flow oxygen and noninvasive ventilation, avoiding tracheal stenting despite severe airway compromise.
- Rapid and dramatic tumor regression was achieved with combined chemo-immunotherapy (nanoparticle paclitaxel, carboplatin, 5-FU, and toripalimab).
- Despite initial response, the disease later progressed with hepatic, pleural, and skeletal metastases, underscoring its aggressive biology and the need for vigilant surveillance and molecularly guided future therapies.

While surgery is definitive for resectable disease, systemic therapy plays a crucial role in unresectable or emergent settings, particularly when airway obstruction limits intervention. Recent evidence supports combining chemotherapy with immune checkpoint inhibitors (ICIs) to achieve rapid control, even in PD-L1–low tumors^[6–8]^.

This case report has been reported in line with the SCARE checklist^[9]^.

## Case report

A 43-year-old male initially developed a gradual change in voice associated with progressive swelling in the right supraclavicular region. Over the following weeks, he began experiencing worsening breathlessness, which later became severe and was accompanied by stridor. On examination, he had a firm right supraclavicular lymph node and suprasternal fullness. MRI of the neck revealed a large tracheal mass arising from the right lateral tracheal wall, measuring 7.9 × 6 × 6 cm, extending from the lower cervical to upper thoracic levels, infiltrating and displacing the trachea. A separate right supraclavicular mass was also identified.

**Figure 1.**
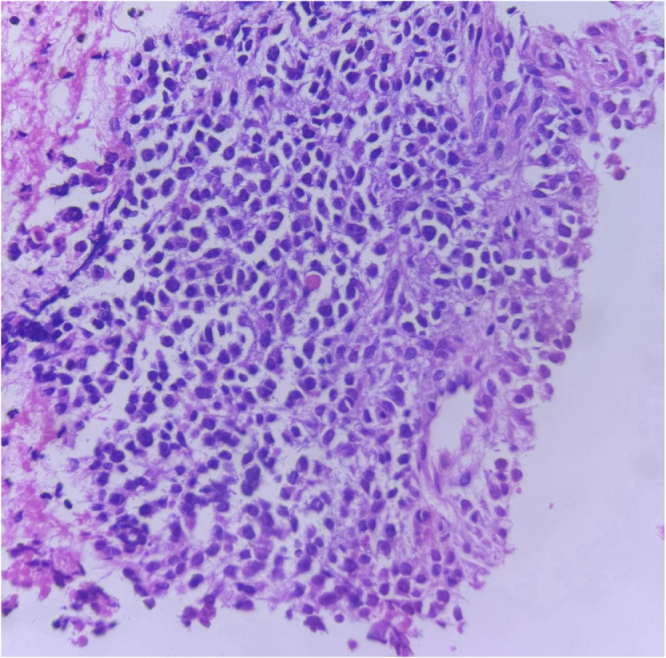
Histopathological section of the supraclavicular lymph node core biopsy showing sheets of poorly differentiated malignant epithelial cells with hyperchromatic pleomorphic nuclei, high nuclear-to-cytoplasmic ratio, and brisk mitotic activity (H&E, ×40). Features were consistent with metastatic poorly differentiated squamous cell carcinoma.

**Figure 2.**
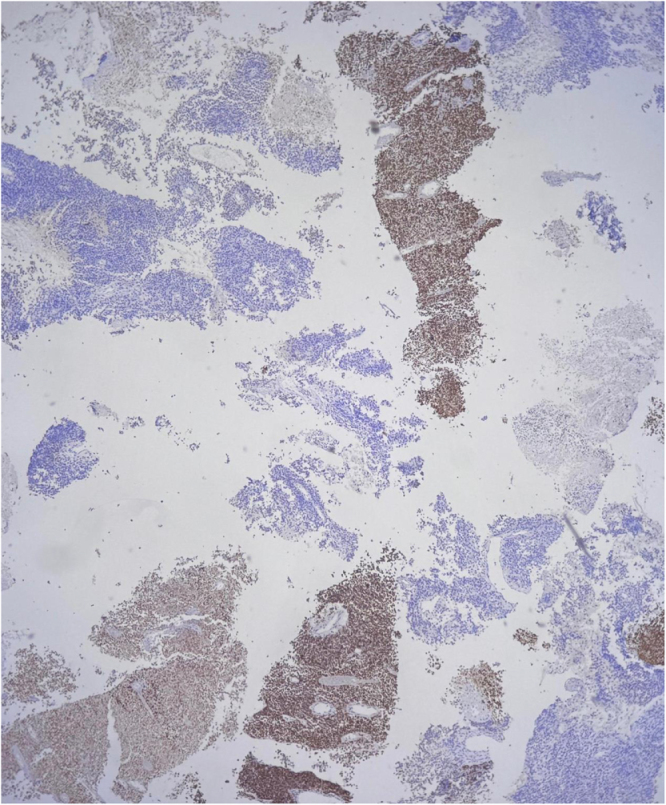
Immunohistochemistry revealing aberrant cytoplasmic positivity for CK20 in tumor cells, an unusual finding in SCC.

**Figure 3.**
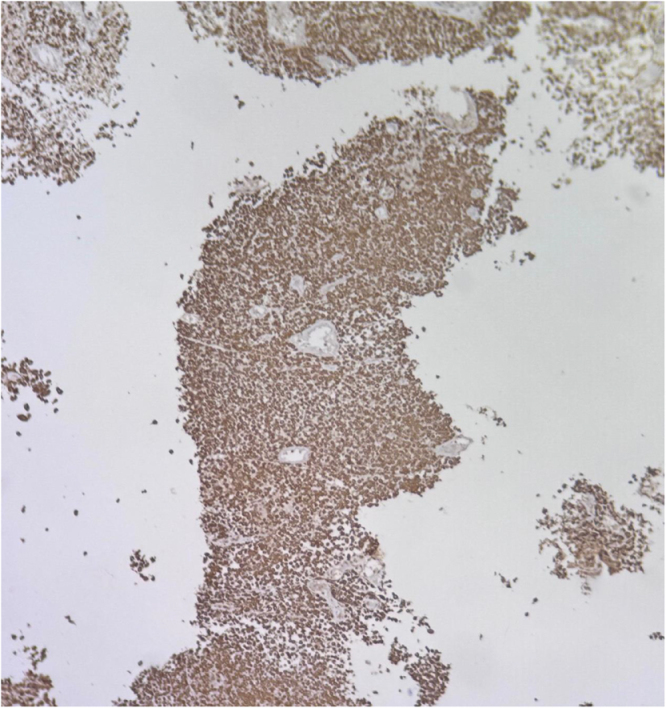
Immunohistochemistry showing diffuse strong cytoplasmic positivity for Pan-Cytokeratin (AE1/AE3) in tumor cells, confirming epithelial origin of the malignant cells.

**Figure 4.**
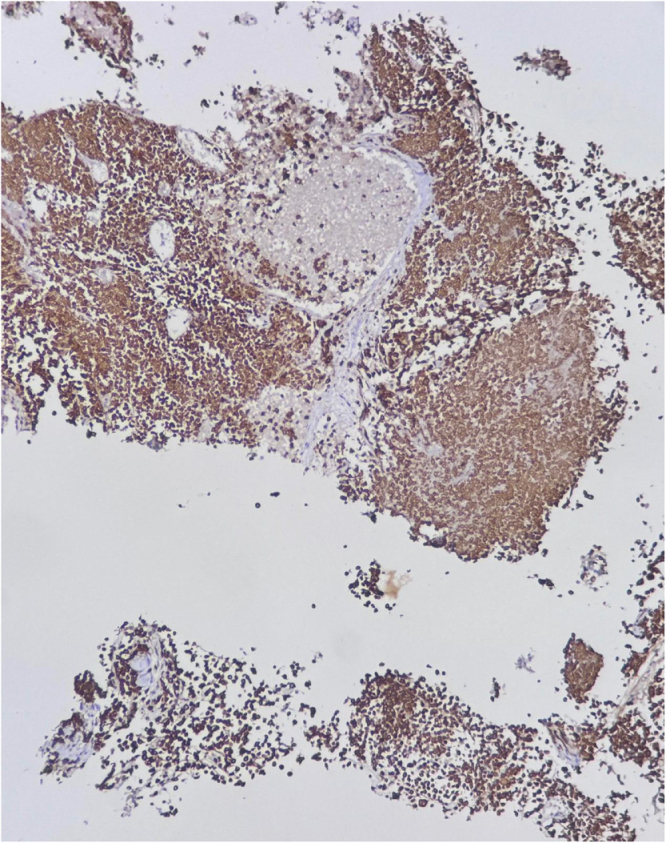
Immunohistochemistry showing strong cytoplasmic and membranous positivity for CK5/6 in tumor cells, supporting squamous differentiation.

**Figure 5.**
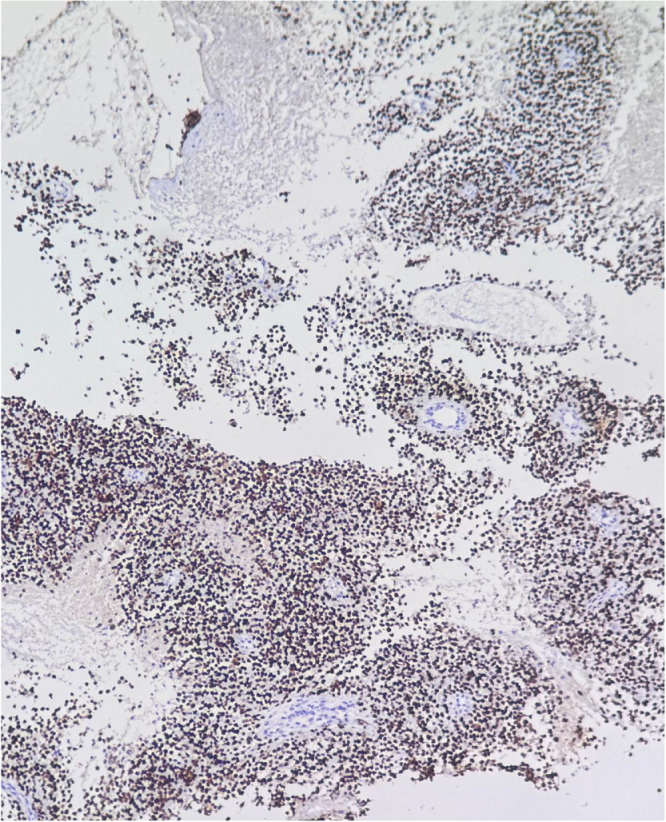
Immunohistochemistry demonstrating diffuse nuclear positivity for p63 in tumor cells, further confirming squamous differentiation.

**Figure 6.**
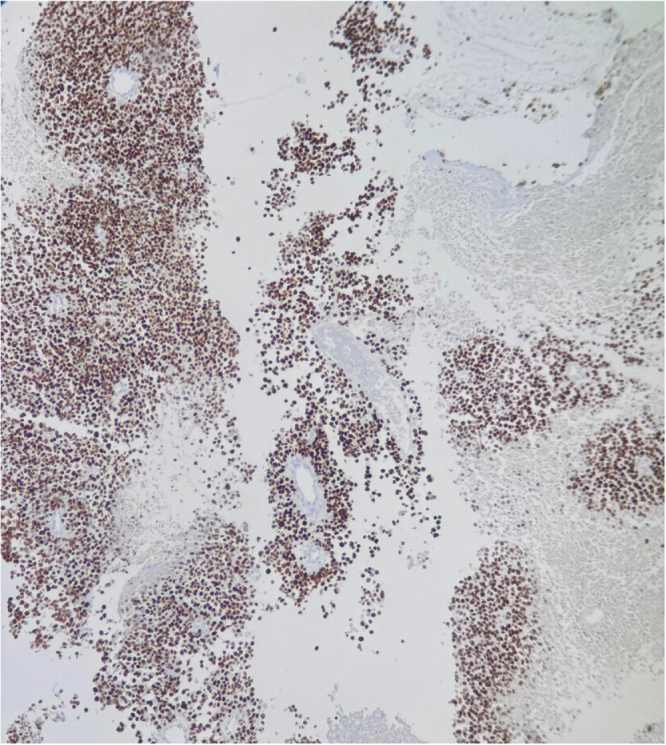
Immunohistochemistry showing diffuse strong nuclear positivity for p40 in tumor cells, consistent with SCC.

Due to increasing respiratory distress, he was admitted to the ICU and supported with noninvasive ventilation (NIV) and high-flow oxygen. Although tracheal stenting was considered, he maintained stable airway pressures and adequate saturation on NIV, allowing invasive airway procedures to be avoided. Core needle biopsy of the right supraclavicular node showed metastatic carcinoma (Fig. 1). Immunohistochemistry confirmed poorly differentiated SCC with aberrant CK20 expression (Fig. 2), positive for AE1/AE3 (Fig. 3), CK5/6 (Fig. 4), p63 (Fig. 5), and p40 (Fig. 6), and negative for neuroendocrine, thyroid, and urothelial markers. PD-L1 expression was low (combined positive score <1), and lactate dehydrogenase (LDH) was elevated. A biopsy of the primary tracheal tumor was not attempted because instrumentation carried a significant risk of precipitating complete airway obstruction in an already compromised airway. Therefore, the supraclavicular node was used for diagnosis.

Once his airway stabilized, systemic therapy was initiated urgently. He received two cycles of taxotere (docetaxel), platinol (cisplatin), and fluorouracil (5-FU)-based chemotherapy consisting of nanoparticle paclitaxel (130 mg), carboplatin (cumulative 1800 mg), and 5-fluorouracil (1250 mg). His stridor gradually improved, and he became clinically stable. During this period, he developed swelling of the left upper limb, venous Doppler imaging confirmed thrombosis of the cephalic and axillary veins, and anticoagulation was started.

He then proceeded to Cycle 3, which included nanopaclitaxel (180 mg), carboplatin (250 mg), 5-fluorouracil (1700 mg), and the addition of toripalimab (240 mg). A positron emission tomography/computed tomography (PET-CT) performed after three cycles demonstrated a marked radiological response, with near-complete resolution of the tracheal lesion and significant reduction of the supraclavicular mass.

He subsequently received Cycle 4, comprising nanopaclitaxel (150 mg), carboplatin (150 mg), and 5-fluorouracil, with dose reductions based on tolerance. Following this, he was planned for concurrent chemoradiotherapy (CCRT). As part of this combined treatment, he received Cycle 6, containing paclitaxel (100 mg) and carboplatin (150 mg) administered alongside radiotherapy.

**Figure 7.**
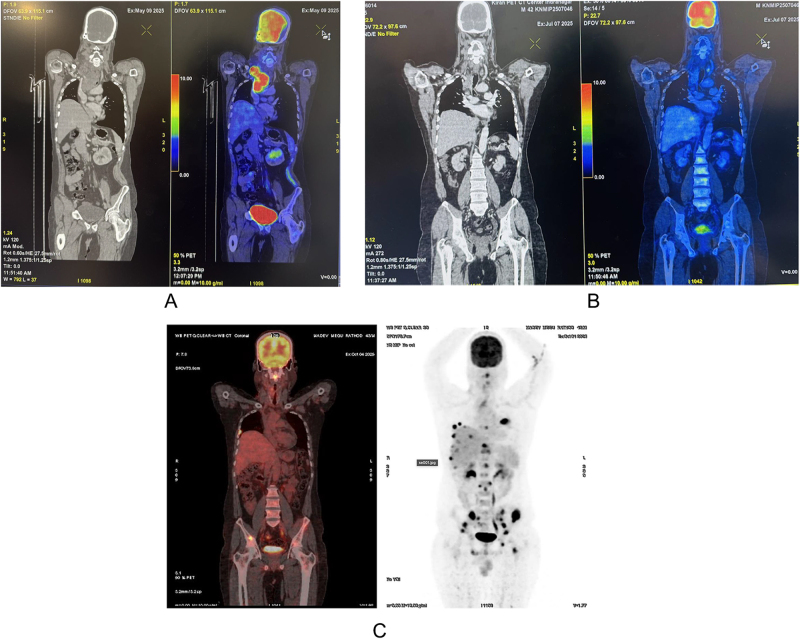
PET-CT evaluation of superior mediastinal SCC response to treatment. (A) Baseline PET-CT scan (before treatment) demonstrates an intensely FDG-avid lesion in the superior mediastinum, consistent with metabolically active nodal disease. (B) Follow-up PET-CT scan (after initial chemo-immunotherapy) shows near-complete resolution of FDG uptake in the mediastinum, suggestive of significant therapeutic response. (C) Latest PET-CT scan demonstrates multiple new FDG-avid hepatic, pleural, and skeletal lesions, indicating systemic metastatic progression despite prior locoregional response.

Despite this strong initial response, he later developed escalating bone pain. Repeat PET-CT imaging revealed new fluorodeoxyglucose (FDG)-avid lesions in the liver, pleura, vertebrae, and pelvis, along with a metabolically active plaque-like lesion along the lateral tracheal wall, indicating aggressive systemic progression after six cycles of systemic therapy (Fig. 7).

**Figure 8.**
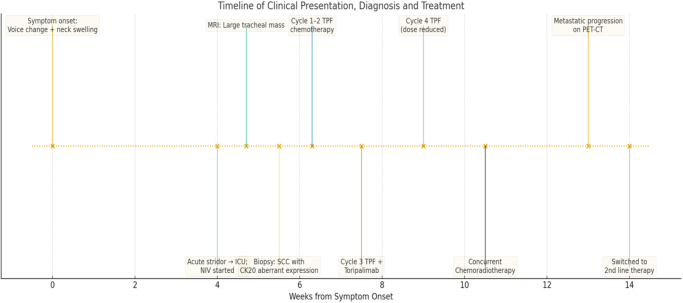
Timeline summary from onset of initial symptoms to systemic progression and treatment modification in a patient with primary tracheal SCC presenting with acute airway obstruction.

In view of this progression, the multidisciplinary team reevaluated his treatment plan and transitioned him to biweekly docetaxel and cisplatin as a second-line regimen (Fig. 8). Prognosis, disease behavior, and options were discussed extensively with the patient and his family.

## Discussion

Primary tracheal SCC is a rare and aggressive malignancy, with limited data guiding optimal management. Gaissert emphasized that early recognition is critical due to the risk of acute airway compromise, noting that delays in diagnosis are common given the nonspecific nature of early symptoms^[1]^. Similarly, Honings *et al*. highlighted that most patients present with locally advanced disease, often with cervical or mediastinal lymph node involvement, which complicates both diagnostic and therapeutic strategies^[2]^. In our patient, presentation with acute stridor and a palpable supraclavicular node reflects this aggressive clinical course.

Aberrant CK20 expression in SCC, though uncommon, is increasingly recognized as a potential diagnostic pitfall. Moll *et al* and Dum *et al* have documented CK20 positivity in nongastrointestinal tumors, including rare cases of SCC, emphasizing the importance of broad immunohistochemical panels to rule out metastatic disease from the gastrointestinal tract or urothelial tract^[3,4]^. Our case further underscores the need to interpret CK20 in the context of other markers, AE1/AE3, CK5/6, p63, and p40 positivity, alongside TTF1, CD5, CD117, GATA3, and LCA negativity, supported the diagnosis of primary tracheal SCC^[1,2,4,5]^.

Acute airway compromise in tracheal malignancies remains a significant challenge. In our patient, NIV and high-flow oxygen were sufficient to stabilize the airway, allowing avoidance of tracheal stenting and enabling prompt chemotherapy initiation. Oyake *et al* described multidisciplinary approaches, including ECMO and airway stenting, for anterior mediastinal masses causing obstruction, highlighting that systemic therapy can sometimes serve as a temporizing measure when procedural interventions are not feasible^[10]^. In our patient, urgent systemic chemotherapy and subsequent addition of toripalimab rapidly relieved airway obstruction, allowing safe continuation of treatment.

The response to chemo-immunotherapy aligns with observations in thoracic malignancies with rare histologies. Gandhi *et al* demonstrated that pembrolizumab combined with platinum-based chemotherapy produced substantial tumor regression even in PD-L1 low nonsmall cell lung cancer^[6]^. Andrini *et al* reported similar benefits in rare thoracic tumors, suggesting that ICIs can be effective irrespective of PD-L1 status^[7]^. Despite this initial response, our patient developed hepatic, skeletal, and pleural metastases, indicating aggressive systemic disease. Arbour and Riely noted that poorly differentiated SCCs of thoracic origin often exhibit early dissemination despite local control, reinforcing the need for vigilant post-treatment surveillance^[11]^.

Recent literature also emphasizes the evolving role of molecular profiling and novel therapeutic strategies in overcoming treatment resistance. Mountzios *et al* highlighted the potential of targeting alternative molecular pathways beyond conventional chemo-immunotherapy to manage thoracic tumors that exhibit rapid progression^[8]^. While such strategies remain investigational, they may offer future options for rare tracheal malignancies with aggressive behavior.

Overall, this case adds novel insights to the literature by documenting a CK20-positive, primary tracheal SCC presenting with acute airway compromise, demonstrating the feasibility and efficacy of rapid chemo-immunotherapy intervention, and highlighting the limitations of current therapy due to early systemic progression. These observations underscore the importance of individualized, multidisciplinary management, early intervention in airway compromised patients, and consideration of molecularly guided therapeutic approaches in rare thoracic malignancies.

## Conclusion

Primary tracheal SCC with aberrant CK20 expression is a rare and aggressive malignancy that can present with acute airway obstruction. Early initiation of chemo-immunotherapy can induce rapid responses and may allow avoidance of tracheal stenting if airway stability is maintained on NIV or high-flow oxygen. However, aggressive biological behavior necessitates close surveillance for early detection of systemic progression. Comprehensive immunoprofiling and consideration of molecular studies may guide future therapeutic strategies, emphasizing the need for individualized, multidisciplinary approaches in managing rare tracheal malignancies.

## Learning points

- Primary tracheal SCC with CK20 aberrant expression is rare and biologically aggressive.
- Tracheal stenting may be avoided if the patient maintains saturation on NIV or high-flow oxygen until chemotherapy initiation.
- Chemo-immunotherapy can produce rapid airway relief and tumor regression, even in PD-L1 low tumors.
- Aggressive disease biology warrants vigilant follow-up and exploration of molecularly guided therapeutic options.

## Patient perspective

The patient expressed relief that the cause of his airway obstruction was promptly identified and managed without the need for tracheal stenting. He was particularly appreciative of the rapid improvement in breathing after initiation of chemotherapy and immunotherapy. Although later saddened by disease progression, he was satisfied with the multidisciplinary care and clear communication provided throughout his treatment course.

## Data Availability

Data supporting the findings of this case report are stored by the corresponding author and are available upon reasonable request.

## Research registration unique identifying number (UIN)

This case report was not eligible for research registration.
